# Intravitreal Injection of Ranibizumab and *CTGF* shRNA Improves Retinal Gene Expression and Microvessel Ultrastructure in a Rodent Model of Diabetes

**DOI:** 10.3390/ijms15011606

**Published:** 2014-01-22

**Authors:** Bojie Hu, Yan Zhang, Qing Zeng, Qian Han, Lijuan Zhang, Mian Liu, Xiaorong Li

**Affiliations:** Tianjin Medical University Eye Hospital/Eye Institute, Fukang Rd. 251#, Nankai Dist., Tianjin 300384, China; E-Mails: hbj151617@163.com (B.H.); yanzhang9927@163.com (Y.Z.); m18450073628@163.com (Q.Z.); hanqian0131@sina.com (Q.H.); zhanglj2004@163.com (L.Z.); tianshi0530@163.com (M.L.)

**Keywords:** vascular endothelial growth factor, connective tissue growth factor, diabetic retinopathy, ranibizumab, shRNA

## Abstract

Therapeutic modalities targeting vascular endothelial growth factor (VEGF) have been used to treat neovascularization and macular edema. However, anti-VEGF treatment alone may cause up-regulation of connective tissue growth factor (CTGF) in the retina, increasing the risk of fibrosis and tractional retinal detachment. Therefore, in this study, we employ a novel dual-target intervention that involves intravitreal injection of the VEGF inhibitor ranibizumab and a transfection reagent-treated non-viral vector carrying anti-*CTGF* short hairpin RNA (shRNA) driven by human RNA polymerase III promoter U6. The effects of the dual-target intervention on the expression of *VEGF* and *CTGF* and on microvessel ultrastructure were examined in retina of streptozocin-induced diabetic rats. *CTGF* was significantly up-regulated at week 8 after diabetic induction, whereas *VEGF* was not up-regulated until week 10. The high expression of both genes was maintained at week 12. Transmission electron microscopy also revealed progressive exacerbation of microvessel ultrastructure during the same period. In addition, ranibizumab significantly lowered *VEGF* but elevated *CTGF* mRNA, whereas *CTGF* shRNA significantly reduced the mRNA levels of both *CTGF* and *VEGF* in diabetic retinas. Importantly, dual-target intervention normalized the transcript levels of both target genes and ameliorated retinal microvessel ultrastructural damage better than either single-target intervention. These results suggest the advantages of dual-target over single-target interventions in diabetic retina and reveal a novel therapeutic modality for diabetic retinopathy.

## 1. Introduction

Diabetic retinopathy (DR) is one of the most common complications associated with diabetes and is the leading cause of blindness in the working-age population. DR is divided into the early phase of non-proliferative diabetic retinopathy (NPDR) and the advanced phase of proliferative diabetic retinopathy (PDR). NPDR is characterized by microvessel endothelial cell apoptosis, pericyte loss, acellular capillary formation, thickening of capillary basement membrane, and breakdown of the blood retina barrier, whereas PDR is characterized by macular edema, neovascularization, retinal hemorrhage and fibrosis, and even retinal detachment [1]. DR has been considered a multifactorial disease. Several pathogenic factors, including pro-inflammatory molecules [2,3], reactive oxygen species [4], diacylglycerol-protein kinase C activation [5], poly-adenosine diphosphate ribose polymerase activation [6], and the polyol pathway [7], are involved in the early phase of the disease. A variety of cytokines and growth factors promote the progression from NPDR to PDR and exacerbate the ongoing PDR conditions [8]. Among these pathogenic factors, vascular endothelial growth factor (VEGF) and connective tissue growth factor (CTGF) are important in both early- and advanced-stage DR [9].

The VEGF protein family includes VEGF-A, placental growth factor, VEGF-B, VEGF-C, VEGF-D, VEGF-E, and snake venom VEGF. VEGF-A has at least nine subtypes, among which VEGF-165 is the most abundant and plays a major role in promoting angiogenesis and vascular permeability [10]. The role of VEGF in DR is well recognized. The up-regulation of VEGF has been detected in the ocular tissues of patients and animals models during the early phase of DR, serving as a microvascular permeability-promoting and proinflammatory factor in retina; moreover, VEGF is elevated in vitreous fluid of PDR patients, contributing to retinal neovascularization and macular edema in the advanced phase of the disease. On the other hand, the inhibition of VEGF by monoclonal antibodies has been widely used and proven effective in reducing retinal neovascularization and macular edema [11–13].

CTGF is a cysteine-rich protein of the CCN family [14]; CCN is an acronym that stands for the first three proteins discovered in this family, cysteine-rich angiogenic protein 61 (CRP61), CTGF, and nephroblastoma overexpressed (NOV). CTGF exerts pro-angiogenic effects by promoting differentiation of CD34^+^ progenitor cells to endothelial cells [15] and stimulating migration, proliferation, and tube formation of endothelial cells [16]. In addition, CTGF is downstream of transforming growth factor-β (TGF-β) and mediates its pro-fibrotic effects. CTGF is up-regulated during fibrosis of several tissues, including lung, kidney and heart [17,18]. In the eye, CTGF is present in tears, aqueous humor, and vitreous fluid [19,20] and is essential to the metabolism and normal function of ocular tissue, whereas under pathological conditions, CTGF concentration in the vitreous of PDR patients is significantly greater than in NPDR patients and normal subjects, and its concentration is positively correlated with PDR stage [21]. Significant elevation of CTGF protein is also found in the retina of diabetic rats [22]. Therefore, results from cell culture, diabetic patients and animal models suggest that CTGF is involved in the progression of DR by increasing the tendency toward angiogenesis and fibrosis.

VEGF and CTGF are key pathogenic factors during the initiation and progression of DR, so they are attractive molecular targets for therapy. Anti-VEGF therapy has been used to treat neovascular diseases in the eye. Indeed, neovascularization and macular edema are decreased following intravitreal injection of the off-label drug bevacizumab (a VEGF monoclonal antibody with the trade name of Avastin), which was originally approved for colon cancer treatment. However, increased retinal fibrosis [23,24] and tractional retinal detachment [25–27] have been reported in the PDR patients following administration of bevacizumab despite neovascular inhibition. Moreover, we have found that PDR patients treated by vitrectomy after intravitreal anti-VEGF treatment tended to have lower VEGF but higher CTGF than those without previous anti-VEGF treatment [28]. These results suggest that single-target intervention with an anti-VEGF monoclonal antibody may induce up-regulation of CTGF, increasing the fibrotic tendency in diabetic retina. Therefore, we hypothesize that a dual intervention that targets both VEGF and CTGF may inhibit the up-regulation of both genes and hence generate beneficial effects on microvessels in diabetic retina. To test this hypothesis, we employed another anti-VEGF agent, ranibizumab (trade name Lucentis), which specifically binds VEGF-A. Ranibizumab was originally designed for ocular application and was approved by the U.S. Food and Drug Administration (FDA) for the treatment of wet age-related macular degeneration (AMD) on 30 June 2006 and for the treatment of diabetic macular edema (DME) on 10 August 2012 [29]. We designed a short hairpin RNA (shRNA) that specifically targets the rat *CTGF* transcript, and cloned the shRNA into a non-viral eukaryotic expression vector downstream of the human RNA polymerase III promoter U6. After testing the efficacy of the anti-*CTGF* shRNA in cells overexpressing rat *CTGF*, ranibizumab and the transfection reagent-treated vector encoding *CTGF* shRNA were injected into the vitreous of the rats at week 10 after diabetic induction. One week later, the distribution of the vector and the expression of the shRNA in retina were observed by visualizing the red fluorescence protein (RFP) that had been cloned into the vector. The expression of the *VEGF* and *CTGF* genes, as well as the microvascular ultrastructure in retina, was also examined. This study suggests a therapeutic target besides VEGF and a novel dual-target intervention modality for DR.

## 2. Results

### 2.1. Blood Glucose Level of Streptozocin-Induced Diabetic Rats

**Figure 1.**
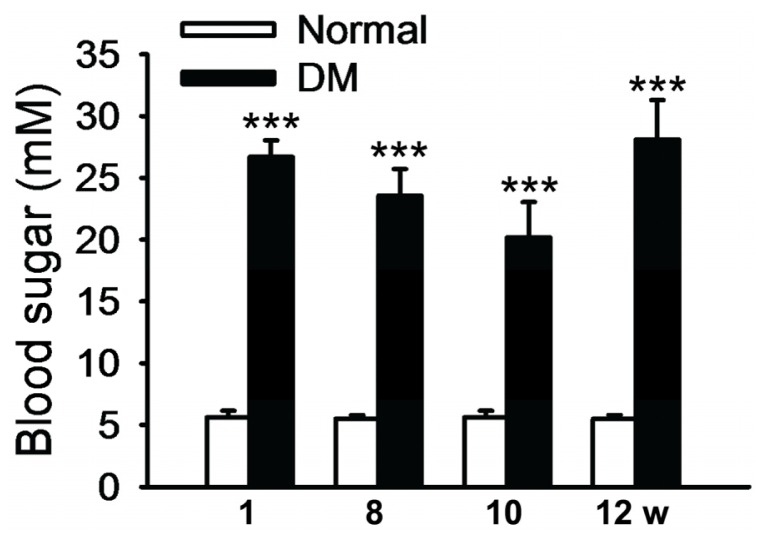
Blood glucose in STZ-induced diabetic rats. Blood glucose in the normal and diabetes mellitus (DM) groups was examined at weeks 1, 8, 10, and 12 following diabetic induction. The data represent mean ± SE, *n* = 5–14, *** *p* < 0.001, Normal *vs.* DM.

Diabetic rats exhibited reduced body weight and symptoms of polydipsia, polyphagia, and polyuria after two weeks of intravenous injection of streptozocin (STZ). The blood glucose of the diabetic animals at weeks 1, 8, 10, and 12 following diabetic induction were 26.69 ± 4.81, 23.56 ± 4.81, 20.19 ± 7.55, and 28.10 ± 5.52 mM, respectively. No statistical significance was found among the blood glucose levels in diabetic rats at these time points, except that the blood glucose level at week 10 was significantly lower than week 1 (*p* = 0.03, Figure 1). More importantly, the blood glucose levels in diabetic rats were all significantly higher than their normal counterparts (*p* < 0.001, Figure 1). These results suggest that intravenous injection of STZ induced the metabolic disorders characterized by high blood glucose, mimicking the symptoms of type I diabetes.

### 2.2. Ultrastructural Changes in Retinal Microvessel of Diabetic Rats

**Figure 2.**
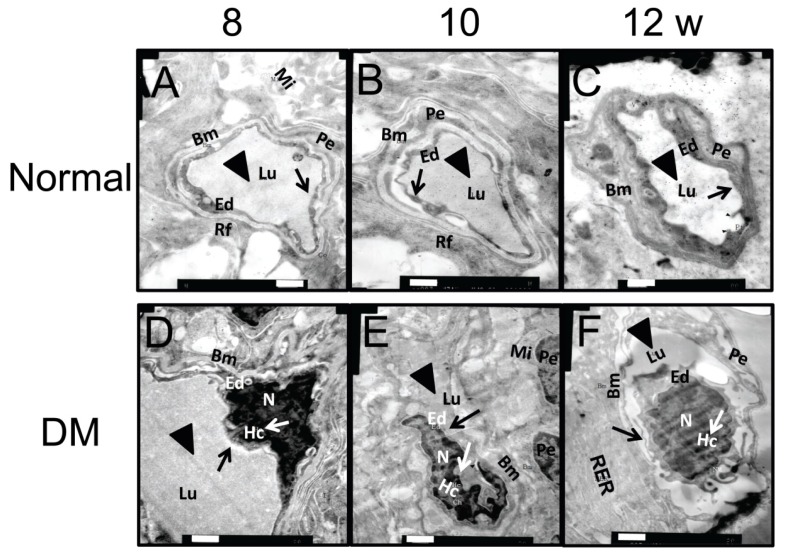
Ultrastructures of retinal microvessels in STZ-induced diabetic rats. The ultrastructures of retinal microvessels in normal (**A**–**C**) and DM rats (**D**–**F**) were examined by TEM at weeks 8, 10, and 12 following diabetic induction. Representative pictures are shown (*n* = 5). Black triangles refer to capillary lumen, black arrows refer to endothelial cells, and white arrows indicate heterochromatin. Bm: Basement membrane, Lu: capillary lumen, Ed: endothelial cell; Pe: pericyte; Mi: mitochondria, Rf: reticular fiber, N: nucleus, Hc: heterochromatin, RER: rough endoplasmic reticulum. Scale bar = 500 nm.

Transmission electron microscopy (TEM) was employed to detect the early changes in retinal microvessels in this rat model of diabetes. At week 8 after diabetic induction, endothelial cell pyknosis and heterochromatin margination (white arrow) were seen. The disruption of basement membrane could be observed sporadically (Figure 2D). As the diabetes progressed to week 10, retinal capillary lumen stenosis (black triangle) and microvessel endothelial cell swelling (black arrow) appeared, while endothelial cell nuclei exhibited a regular shape except for the depressed nuclear membrane (Figure 2E). At week 12 after diabetes induction, thickening of the capillary basement membrane was observed with an increased electron density, which is the typical ultrastructural change in diabetic retina. In addition to the heterochromatin pyknosis (white arrow), cell swelling (black arrow), and lumen stenosis (black triangle) observed at the previous time points, the cell nucleus had a tortuous shape and was heterogeneously swelled (Figure 2F). In contrast, the microvessels of normal retina exhibited an open lumen (black triangles) and continuous basement membrane with uniform electron density at each time point examined (Figure 2A–C). The morphological data suggest that the ultrastructural changes in retinal microvessels were readily detectable by TEM from week 8 to 12 after disease onset in the STZ-induced diabetic rats.

### 2.3. Expression of *VEGF* and *CTGF* Genes in Retina of Diabetic Rats

**Figure 3.**
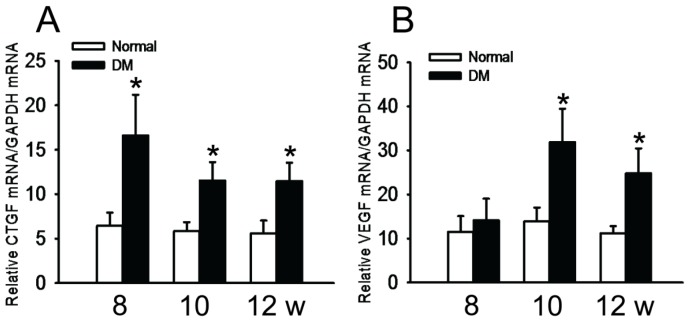
Dynamic changes of *CTGF* and *VEGF* gene expression in retina of diabetic rats. (**A**) Relative *CTGF* mRNA levels in diabetic retinas and normal controls; (**B**) Relative *VEGF* mRNA levels in the retina of diabetic rats and normal controls. The data represent mean ± SE, *n* = 6, * *p*<0.05, Normal *vs.* DM.

The results of RT-PCR showed that the transcript levels of *CTGF* were significantly higher in diabetic retinas than in normal ones as early as week 8 following STZ induction (*p* < 0.05), and this up-regulation was maintained through week 12 of diabetes (*p* < 0.05) (Figure 3A). On the other hand, the *VEGF* mRNA level in diabetic retina was similar to normal retina (*p* = 0.669) at week 8 following diabetic induction, but it was twice as high as in the normal retina at week 10 after diabetes induction (*p* < 0.05, DM *vs.* Normal), the trend of *VEGF* expression in the diabetic retinas was maintained to week 12 following the disease onset (*p* < 0.05, DM *vs.* Normal) (Figure 3B).

### 2.4. Efficacy of Anti-*CTGF* shRNA

**Figure 4.**
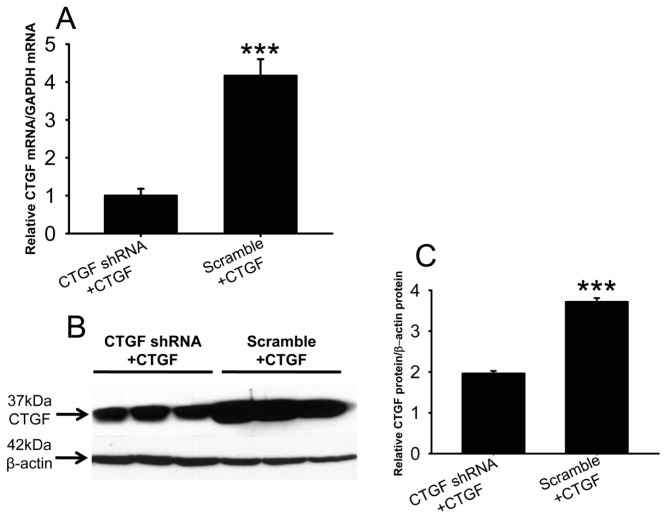
Efficacy of the shRNA against rat *CTGF* in HEK 293 cells overexpressing rat *CTGF*. HEK 293 cells were co-transfected with anti-*CTGF* shRNA or scramble shRNA and rat *CTGF* cDNA. Forty-eight hours post-transfection, real-time PCR (**A**) and western blot (**B**) for *CTGF* were performed. The mRNA and protein levels of *CTGF* were normalized to *GAPDH* and *β-actin*, respectively; The western blot bands were quantified (**C**). The data represent mean ± SE, *n* = 3, *** *p* < 0.001, CTGF shRNA + CTGF *vs.* Scramble + CTGF.

In human embryonic kidney 293 (HEK293) cells co-transfected with *CTGF* shRNA and a *CTGF* expression plasmid, the *CTGF* mRNA levels were only 25% of those in the cells transfected with scramble shRNA and the *CTGF* expression plasmid (Figure 4A, *p* < 0.001, CTGF shRNA + CTGF *vs.* scramble + CTGF). In addition, both CTGF and β-actin protein were detected at the expected positions (Figure 4B), and the amount of CTGF protein in the *CTGF* shRNA cells was approximately 50% of that in the scramble shRNA-treated cells (Figure 4C, *p* < 0.001, CTGF shRNA + CTGF *vs.* scramble + CTGF). These results suggest that the anti-*CTGF* shRNA efficiently knocked down CTGF expression at both the mRNA and protein levels.

### 2.5. Distribution of the Vector Encoding Anti-*CTGF* shRNA in Diabetic Retina

**Figure 5.**
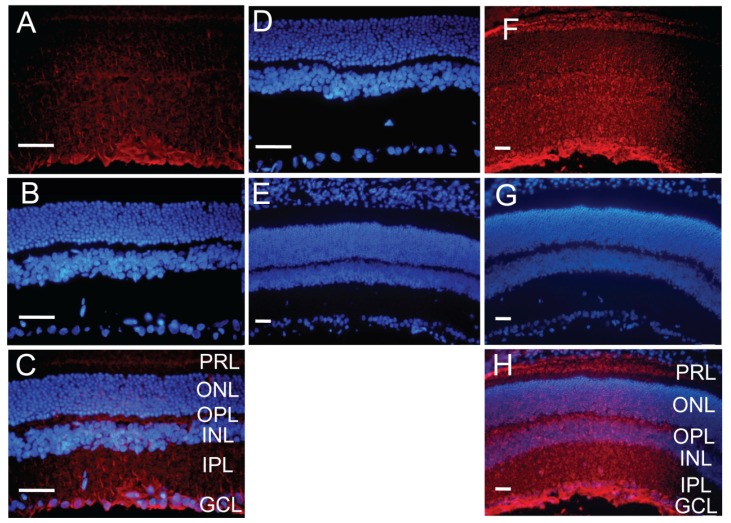
Expression of the vector encoding anti-*CTGF* shRNA in diabetic retinas after intravitreal injection. One week after injection, the retinas of diabetic rats were sectioned, and the expression of RFP from the injected vector was directly visualized (**A**) or detected by immunostaining (**F**) in all layers of retina. DAPI counterstaining (**B** and **G**) and merged pictures (**C** and **H**) are also shown. Retinal sections of the un-injected eyes (**D**) and the eyes injected with the vector without RFP (**E**) served as negative controls, and no RFP signal was visualized or detected in these sections. *n* = 5, PRL: photoreceptor layer, ONL: outer nuclear layer, OPL: outer plexiform layer, INL: inner nuclear layer, IPL: inner plexiform layer, GCL: ganglion cell layer. Scale bar = 50 μm.

One week after injection, the expression of RFP in the anti-*CTGF* shRNA vector was directly visualized across the retina from the ganglion cell layer to the photoreceptor layer (Figure 5A,C). The RFP fluorescence intensity appeared to be higher in inner retina than outer retina. The immunostaining results also showed a similar expression pattern of RFP in all layers of diabetic retina (Figure 5F,H). In contrast, no RFP signal was observed in the un-injected eyes (Figure 5D) or detected by immunostaining in the eyes injected with the vector without RFP (Figure 5E), suggesting that the red fluorescence was not due to autofluorescence or artifacts of intravitreal injection. These results are comparable to the previous report on intravitreal delivery of *CTGF* siRNA [30], and suggest that the transfection and expression of the vector in diabetic retina are feasible.

### 2.6. Effects of Dual-Target Intervention on *VEGF* and *CTGF* Expression in Retina of Diabetic Rats

**Figure 6.**
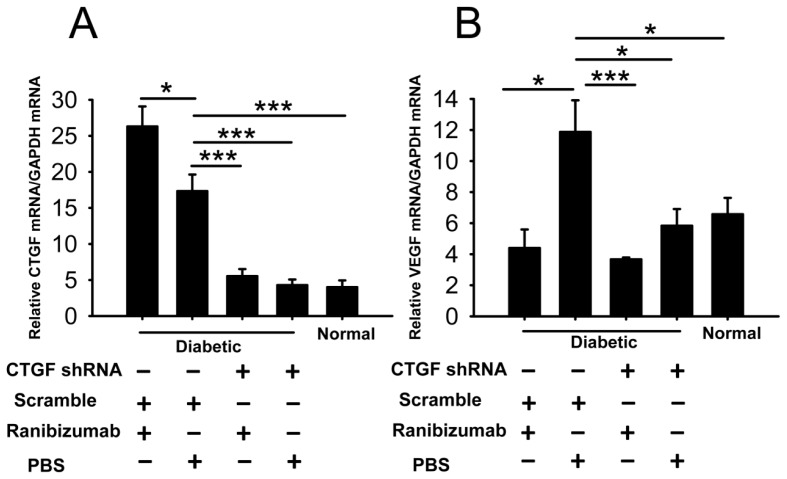
Gene expression changes of *CTGF* and *VEGF* in diabetic retina after dual-target intervention using ranibizumab and *CTGF* shRNA. (**A**) Relative *CTGF* mRNA levels in diabetic and normal retinas; (**B**) Relative *VEGF* mRNA levels in diabetic and normal retinas. The bottom panel indicates the intervention to which the diabetic rats were subjected, + indicates with the intervention, − indicates without. The data represent mean ± SE, *n* = 5, * *p* < 0.05, *** *p* < 0.001.

The real-time PCR results showed that ranibizumab single-target intervention down-regulated *VEGF* mRNA in diabetic retina similar to that in normal controls (*p* > 0.05, ranibizumab + scramble *vs.* normal). In retina of ranibizumab-treated diabetic rats, *VEGF* expression was only 30% of that detected in PBS-treated diabetic retina (*p* < 0.05, ranibizumab + scramble *vs.* PBS + scramble) (Figure 6B). However, ranibizumab single-target intervention caused significant up-regulation of *CTGF* mRNA compared to that in the PBS-treated diabetic retina (*p* < 0.05, ranibizumab + scramble *vs.* PBS + scramble, Figure 6A). This suggests that blockade of VEGF signaling by ranibizumab may induce a reactive up-regulation of CTGF. On the other hand, in the *CTGF* shRNA single-target intervention group, *CTGF* mRNA level in the diabetic retina was reduced to a level similar to the normal controls (*p* > 0.05, CTGF shRNA + PBS *vs.* normal) (Figure 6A). The level of *VEGF* transcript was also significantly decreased in comparison to the scramble shRNA-treated diabetic group (*p* < 0.05, CTGF shRNA + PBS *vs.* scramble + PBS, Figure 6B). More importantly, in the ranibizumab and *CTGF* shRNA dual-target intervention group, the transcript levels of both *VEGF* and *CTGF* were significantly lower than those in the scramble shRNA and PBS-treated diabetic group (*p* < 0.001, ranibizumab + CTGF shRNA *vs.* PBS + scramble) and were comparable to those in their normal counterparts (*p* = 0.292 for *VEGF*, *p* = 0.287 for *CTGF*, ranibizumab + CTGF shRNA *vs.* normal) and to those in the corresponding single-target intervention group (*p* = 0.515 for *VEGF*, ranibizumab + CTGF shRNA *vs.* ranibizumab + scramble; *p* = 0.342 for *CTGF*, ranibizumab + CTGF shRNA *vs.* PBS + CTGF shRNA) (Figure 6A,B). These results suggest that the dual-target intervention can efficiently down-regulate both target genes.

### 2.7. Effects of Dual-Target Intervention on Ultrastructural Changes in Retina of Diabetic Rats

**Figure 7.**
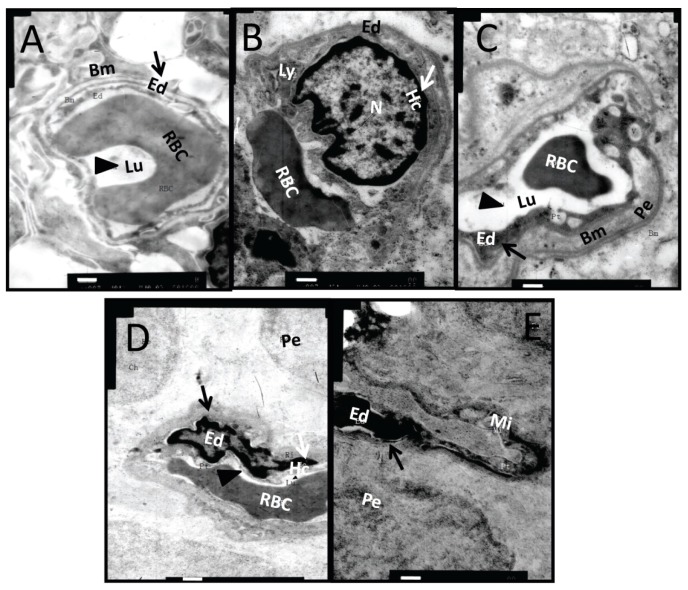
The ultrastructural changes in retinal microvessels of diabetic rats after dual-target intervention. One week after intervention, the ultrastructure changes of retinal microvessels were examined by TEM in normal controls (**A**); diabetic rats without intervention (**B**); diabetic rats subjected to ranibizumab and *CTGF* shRNA dual-target intervention (**C**); and diabetic animals subjected to ranibizumab (**D**) or *CTGF* shRNA (**E**) single-target intervention. Representative pictures are shown (*n* = 5). Black triangles indicate capillary lumen, black arrows indicate endothelial cells, and white arrows indicate heterochromatin. Bm: Basement membrane, Lu: capillary lumen, Ed: endothelial cell; Ly: lysosomes; Pe: pericyte; Mi: mitochondria, N: nucleus, Hc: heterochromatin, RBC: red blood cell. Scale bar = 500 nm.

**Figure 8.**
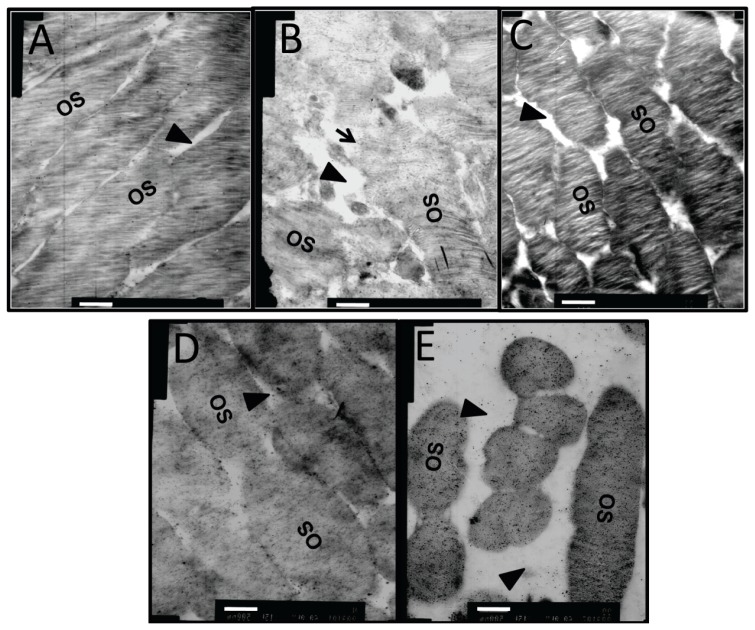
The ultrastructural changes in the retinal photoreceptor outer segment of diabetic rats after dual-target intervention. Representative TEM images of retinal photoreceptor outer segments in normal controls (**A**); diabetic rats without intervention (**B**); diabetic rats with dual intervention (**C**); and diabetic animals with ranibizumab (**D**) or *CTGF* shRNA single intervention (**E**) are shown (*n* = 5). Black triangles designate the gap between outer segments, black arrow shows the dissolved membrane discs. OS: outer segment. Scale bar = 500 nm.

In the retina of normal controls (Figure 7A), the capillary lumen (black triangle) was smooth and intact, with red blood cells passing through. The thin sheath of endothelial cells (black arrow) closely attached to the continuous base membrane. The photoreceptor outer segments (OS) were intact and organized with little space (black triangle) between them (Figure 8A). In diabetic retinas without intervention, heterochromatin margination (white arrow), kidney-shaped swelled nucleus, and multiple secondary lysosomes were observed in microvessel endothelial cells (black arrow), and retinal capillary lumen was narrowed and ill-defined (Figure 7B). The organization of OS was disrupted, the gap between OS became larger (black triangle), and some structures of the OS were blurred due to breakage and dissolution of membrane discs (black arrow) (Figure 8B). Notably, the capillary lumen (black triangle) in retina of the dual-target intervention group (Figure 7C) was unobstructed, in which passing red blood cells were seen. Endothelial cells (black arrow) in this group were slightly swelled, and pericytes were clear and intact (Figure 7C). Moreover, photoreceptor OS in this group was organized with a discernible structure of the membrane discs, and the inter-OS space was much reduced compared to that in diabetic retina without intervention (black triangle) (Figure 8C). In contrast, stenotic retinal capillary lumen (black triangle) and deformed red blood cells were observed in both single-target intervention groups (Figure 7D,E). Swelled endothelial cells (Figure 7D,E, black arrow), marginalized heterochromatin (Figure 7D,E, white arrow), and loosely organized photoreceptor OS with a blurred membrane disc structure (Figure 8D,E) were also found in the retina of these groups. The TEM results indicate that the ultrastructure of retinal microvessels and photoreceptor OS in the dual-target intervention group were less damaged than the single-target and non-intervention groups, suggesting the greater effectiveness of the dual-target intervention on diabetic retina.

## 3. Discussion

The application of anti-VEGF drugs for PDR is a relatively new biological treatment modality, and it has been reported to improve vision in DR patients [31–33]. Similarly, our previous clinical study showed that after intravitreal injection of anti-VEGF agents in PDR patients with fibrovascular membrane, the protein level of VEGF in the fibrovascular membrane was significantly reduced. However, the level of CTGF was significantly elevated, and the fibrovascular membrane tended to grow larger [28]. Others also reported that following intravitreal injection of VEGF monoclonal antibody, the balance between VEGF and CTGF was shifted, and an “angiofibrotic switch” was turned on to favor a fibrotic phase in the PDR patients. Therefore, fiber traction components were augmented in the fibrovascular membrane, and retinal fibrosis was accelerated in these patients [23,24]. Yet a similar phenomenon was also observed in patients with wet AMD following anti-VEGF treatment [34]. Therefore, we assessed both VEGF and CTGF as intervention targets in this study to antagonize the pro-angiogenic and pro-fibrotic tendency in retina of STZ-induced diabetic rats. We employed ranibizumab, a monoclonal antibody to VEGF that has recently been approved by the FDA to treat wet AMD and DME, and a shRNA specifically against *CTGF* mRNA to inhibit VEGF signaling and *CTGF* expression, respectively. The dual-target intervention resulted in highly significant reduction of both *VEGF* and *CTGF* transcripts in diabetic retinas (Figure 6A,B). In contrast, the single-target anti-VEGF intervention caused inhibition of *VEGF* expression and concomitant up-regulation of *CTGF* (Figure 6A,B), which were consistent with our clinical observations on the protein expression of both factors [28]. Furthermore, the ultrastructure of retinal microvessels in the dual-target intervention group exhibited a relatively unobstructed capillary lumen, slightly swelled endothelial cells and clear pericytes, outcomes that were superior to the single-target intervention groups and were similar to the morphology in normal retinas (Figure 7). The results of gene expression and TEM analyses suggest that the dual-target intervention might be a novel and effective supplement to the current anti-VEGF modality for DR.

**Figure 9.**
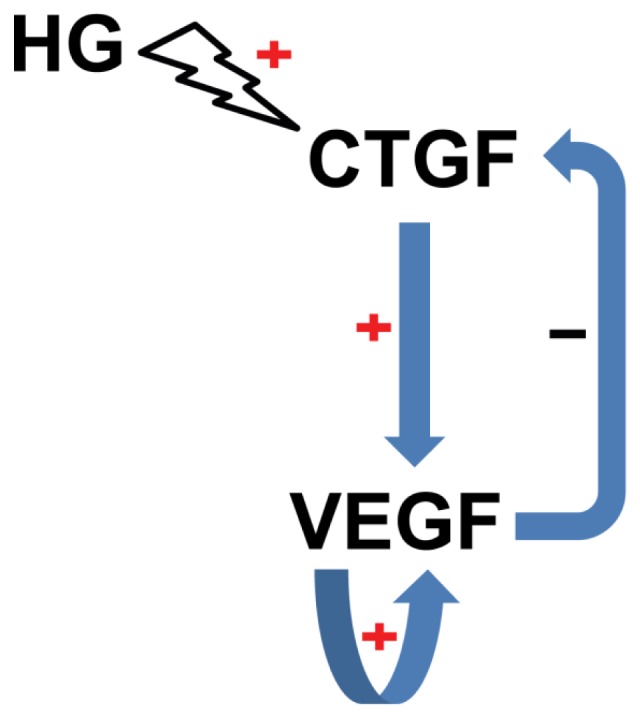
The proposed model for the regulatory network between CTGF and VEGF in the retina of STZ-induced diabetic rats. High glucose up-regulates CTGF, which in turn induces VEGF up-regulation. The increased *VEGF* transcript or VEGF-mediated signaling has a positive-feedback effect on its own expression and a negative-feedback effect on *CTGF* expression.

In addition, the results of the targeted interventions in diabetic retinas shed light on the regulatory mechanisms of *VEGF* and *CTGF* expression. Our gene expression results show that ranibizumab, a monoclonal antibody that blocks VEGF or VEGF-mediated signaling, reduced *VEGF* transcript level in diabetic retinas (Figure 6B). This interesting phenomenon suggests that VEGF may promote its own expression in our experimental paradigm. Indeed, VEGF enhances its own expression in a protein kinase C α-dependent positive-feedback mechanism in human umbilical vein endothelial cells [35]. Therefore, we speculate that as part of the regulatory network of *VEGF* and *CTGF* gene expression, a similar positive-feedback effect on *VEGF* expression might also exist in diabetic retinas (Figure 9). Furthermore, our gene expression analyses showed that the shRNA against *CTGF* resulted in significant reduction of both *CTGF* and *VEGF* transcripts (Figure 6A,B). This shRNA efficiently knocked down *CTGF* expression at both the transcript and protein levels in the cells overexpressing the rat *CTGF* gene (Figure 4A,B) without affecting the levels of internal controls, and we used transfection reagent-facilitated intravitreal delivery. Therefore, the down-regulation of *VEGF* after anti-*CTGF* shRNA administration was likely not due to off-target effects or non-specific activation of the Toll-like receptor [36]. These results instead suggest a mutual regulatory mechanism between *CTGF* and *VEGF* gene expression under DR. The mutual regulatory mechanisms between these two genes in the literature are controversial. Kuiper and colleagues [37] have shown that VEGF induced the expression of *CTGF* and extracellular matrix genes both *in vitro* and *in vivo* and stimulates the secretion of CTGF, thus playing important roles in the induction of DR, fibrosis, and angiogenesis. In contrast, Winkler and coworkers [30] have reported that knocking down *CTGF* expression by *CTGF* siRNA did not affect *VEGF* expression at the transcript level. Yang *et al*. [38] showed that ocular *CTGF* transcript level began to increase at week 8 after diabetes, and the mRNA levels of *VEGF* and *TGF-β2* did not do so until 12 weeks after disease onset. They also showed that the expression of *CTGF*, *VEGF* and *TGF-β2* were all decreased by intravitreal injection of a siRNA targeting *CTGF*. Our results are quite in line with Yang *et al*. [38], as the up-regulation of *CTGF* preceded that of *VEGF* by two weeks (Figure 3) and *CTGF* shRNA knocked down both *VEGF* and *CTGF* (Figure 6) in diabetic retina. These results suggest a leading role of CTGF in the mutual regulatory network of these two pathogenic factors in the retina of diabetic rats (Figure 9). Lastly, the results from our previous [28] and current studies (Figure 6A) and from the literature [23,24] show that VEGF antibody injected into diabetic retinas induced an up-regulation of *CTGF* at either the mRNA or protein level. This may imply a negative-feedback effect between VEGF or VEGF-mediated signaling and *CTGF* expression. Therefore, we propose a model for the mutual regulatory network between *CTGF* and *VEGF* gene expression in diabetic retina. *CTGF* expression is initially up-regulated, which leads to up-regulation of *VEGF*. The increased VEGF or VEGF-mediated signaling, on one hand, self-perpetuates its own expression, and on the other hand, negatively regulates *CTGF* expression (Figure 9). It would be interesting to verify this mutual regulatory network between *VEGF* and *CTGF* and identify the signaling pathway mediating their mutual regulation under diabetic conditions.

The STZ-induced diabetic rat model has been widely used to study pathogenic mechanisms and the effects of potential intervention approaches for DR [30,38,39]. The model exhibits hyperglycemia-induced lesions and inflammation in retinal vascular network and neuroretina in adult animals [40], which is consistent with the pathogenesis and natural history of DR. Therefore, we chose this model to examine the relatively long-term effects of the dual-target intervention on gene expression and retinal ultrastructures. However, this model does not present with retinal neovascularization or fibrosis [40], the important characteristics of DR at the advanced phase. It would also be interesting to test the effects of the dual-target intervention in oxygen-induced retinopathy [41] and the spontaneously diabetic Torii rat [42], the rat models with retinal neovascularization and fibrosis, respectively, to supplement our current results.

It is also worth mentioning that the intravitreal delivery of the vector encoding anti-*CTGF* shRNA was facilitated through the transfection reagent TransIT-TKO^®^ (Mirus Bio LLC, Madison, WI, USA). TransIT-TKO^®^ is a non-liposomal formulation of cationic polymer and lipid that was originally designed for siRNA transfection or DNA and siRNA co-transfection into cultured cells. However, this transfection reagent has been reported to efficiently transfect siRNA and shRNA-encoding vectors into retina [43] and dendritic cells [44,45], respectively. These reports prompted us to explore the *in vitro* and *in vivo* applications of this transfection reagent. Indeed, the efficient knocking down of the *CTGF* overexpression at both mRNA and protein levels in HEK 293 cells co-transfected with *CTGF* shRNA and cDNA encoding vectors (Figure 4A,C) using TransIT-TKO^®^, as well as the widespread expression of RFP across all the retinal layers following intravitreal injection of the TransIT-TKO^®^-treated *CTGF* shRNA vector (Figure 5A–C,F–H), suggests the feasibility of using this transfection reagent for shRNA vector delivery.

In summary, dual-target intervention using ranibizumab and *CTGF* shRNA in the retina of STZ-induced diabetic rats not only reduced the up-regulated target gene expression to a level similar to that in normal controls but also ameliorated the damage of retinal microvessel ultrastructures greater than either single-target intervention. Our results indicate the potential for this dual-target intervention to be developed into a novel therapeutic modality for DR.

## 4. Experimental Section

### 4.1. Animals

One hundred and twenty eight-week-old male Sprague-Dawley (SD) rats with a body weight of 220–250 g were purchased from the Institute of Radiation, Chinese Academy of Medical Sciences (Beijing, China). The animals had free access to food and water and were maintained under a 12 h:12 h light-dark cycle at 22–25 °C. All of the experimental procedures were approved by Institutional Animal Care and Use Committee (IACUC) of Tianjin Medical University (Permit Number: SYXK 2009-0001), and were in accord with the Association For Research In Vision And Ophthalmology Statement for the Use of Animals in Ophthalmic and Vision Research.

### 4.2. Induction of Diabetes

Thirty SD rats were intravenously injected with streptozocin (STZ, Sigma, Shanghai, China, 2% in 0.1 M sodium citrate buffer, pH 4.5) at 45 mg/kg to induce diabetes. The blood glucose and urine sugar levels were measured at 72 h post-injection. Only the rats with blood glucose higher than 16.7 mM were included in the diabetes mellitus group (DM group) for the following experiments. The same number of rats were intravenously injected with sodium citrate buffer (0.1 M, pH 4.5) and served as normal controls.

On weeks 8, 10, and 12 after diabetic induction, retina samples from the DM and normal control groups were quickly dissected, snap-frozen in liquid nitrogen, and stored in −80 °C for gene expression analyses. The eye cups from both groups were collected for TEM analyses.

### 4.3. Design of shRNA against Rat *CTGF*

The shRNA against rat *CTGF* (5′-CACCGCAATACCTTCTGCAGGCTGGATTCAAGAGATCC AGCCTGCAGAAGGTATTGTTTTTTG-3′) was designed according to the rat *CTGF* cDNA sequence (PubMed accession # NM_022266.2), then synthesized and cloned into the multiple cloning sites of a eukaryotic expression vector, GV102 (GeneChem, Shanghai, China). The expression of the cloned *CTGF* shRNA was driven by human RNA polymerase III promoter U6. The coding sequence of green fluorescent protein on GV102 was either replaced by RFP to reduce autofluorescence or removed to generate a negative control. RFP expression was driven by a cytomegalovirus promoter in the vector.

### 4.4. Efficiency of Anti-*CTGF* shRNA in Cells Overexpressing Rat *CTGF*

Human embryonic kidney 293 (HEK 293) cells (Chinese academy of Science, Shanghai, China) were maintained in Dulbecco’s Modified Eagle Medium (Life Technologies, Tianjin, China), 10% Fetal Bovine Serum (Life Technologies, Tianjin, China), 100 U/mL penicillin, 100 μg/mL streptomycin (Life Technologies, Tianjin, China) and 2 mM l-glutamine (Life Technologies, Tianjin, China) at 37 °C and 5% CO_2_ in a humidified incubator. Cells were seeded in 6-well plates at the density of 8 × 10^5^/well. On the next day, the cells were co-transfected with equimolar concentrations of the vector encoding anti-*CTGF* shRNA or scramble shRNA and the expression plasmid containing rat *CTGF* cDNA (Open Biosystems, Thermo Fisher Scientific, Beijing, China) using TransIT-TKO^®^ Transfection Reagent (Mirus Bio LLC, Madison, WI, USA) according to the manufacturer’s protocol. The cells were harvested at 48 h post-transfection for RNA analyses and western blotting. The RNA analyses are described in the following section. The western blotting was performed as previously described [46]. The blot was probed with a rabbit anti-CTGF (polyclonal, 1:500, Santa Cruz Biotechnology, Tianjin, China) and a mouse anti-β-actin (monoclonal, 1:10 000, Sigma, Shanghai, China) and were incubated with horseradish peroxidase-conjugated secondary antibodies. The signals were visualized with enhanced chemiluminescence plus reagents (Amersham Biosciences, Roche, Tianjin, China). The density of the protein bands on the developed image was quantified by Quantity One software (Bio-Rad, Hercules, CA, USA).

### 4.5. Intravitreal Injection of *CTGF* shRNA and Ranibizumab

Fifty rats at week 10 after diabetic induction were subjected to targeted interventions by intravitreally injecting molecules targeting VEGF and/or CTGF. The commercially available ranibizumab was used as an anti-VEGF agent (10 mg/mL, Novartis, Beijing, China), and the anti-*CTGF* shRNA was used to target *CTGF* transcript. Ten normal rats intravitreally injected with phosphate buffer solution (PBS) were also included as normal controls. On the day of injection, the vector carrying either *CTGF* shRNA or scramble shRNA was treated with TransIT-TKO^®^ Transfection Reagent (Mirus Bio LLC, Madison, WI, USA). Briefly, 6 μg vector was incubated with 6 μL TransIT-TKO^®^ Transfection Reagent (Mirus Bio LLC, Madison, WI, USA) at room temperature for 30 min in 50 μL of sterilized H_2_O. The solution was vacuum-evaporated and re-dissolved in 2.5 μL H_2_O. The animals were then anesthetized with intraperitoneal injection of 10% chloral hydrate, the pupils were dilated with tropicamide, and oxybuprocaine hydrochloride was applied to the cornea. The right eyes were injected at 2 mm posterior to the limbus with a 30 G needle. Care was taken to avoid lens damage. The dual-target intervention group received 2.5 μL *CTGF* shRNA-encoding vector:transfection reagent mixture and 2.5 μL ranibizumab (25 μg), whereas the VEGF single-target intervention group received 2.5 μL ranibizumab (25 μg) and 2.5 μL transfection reagent-treated scramble shRNA. For the *CTGF* shRNA single intervention group, 2.5 μL PBS and 2.5 μL transfection reagent-treated *CTGF* shRNA vector were injected. The non-intervened diabetic rats received an injection of 2.5 μL of PBS and 2.5 μL of transfection reagent-treated scramble shRNA. After injection, the needle was kept in the vitreous cavity for 15 s prior to gentle withdrawal. Ofloxacin eye ointment was applied to prevent infection. The fundus of the rats was closely monitored, and the animals with retinal hemorrhage and detachment were excluded. One week after injection, all rats were killed. The retina samples were collected for gene expression, TEM, and fluorescence microscopy analyses.

### 4.6. RNA Extraction and Reverse Transcription

The retina samples were thoroughly grounded in liquid nitrogen with a mortar and pestle and homogenized in lysis buffer. Total RNA was extracted using a GeneJET RNA Purification Kit (Fermentas, Tianjin, China) according to the manufacturer’s protocol. The concentration and purity of total RNA was determined by a Nanodrop 2000 (Thermo Scientific, Tewksbury, MA, USA). Following DNase I digestion, 200 ng total RNA was reverse-transcribed into cDNA in a 20 μL reaction mixture using RevertAid cDNA synthesis Kit (Thermo scientific, Tianjin, China).

### 4.7. Real-Time PCR Analyses for *CTGF* and *VEGF* Expression

Three microliters of cDNA was used as template. The forward primer for the rat *VEGF* gene was 5′-GGGCTCGCGGGATTG-3′; the reverse primer was 5′-GCGCAGACCACGGCTACT-3′. The forward primer for the rat *CTGF* gene was 5′-CCGCCAACCGCAAGATT-3′; the reverse primer was 5′-CACGGACCCACCGAAGAC-3′. The amplification of rat *GAPDH* was also included as internal standard using the forward primer 5′-TGTGTCCGTCGTGGATCTGA-3′ and the reverse primer 5′-CCTGCTTCACCACCTTCTTGA-3′. The 2× universal master mix (FastStart Universal SYBR Green Master, Roche, Tianjin, China) and nuclease-free H_2_O were also added to the reaction mixture. The real-time PCR program consisted of an initial incubation at 50 °C for 2 min and denaturation at 95 °C for 10 min, followed by 40 cycles of denaturation at 95 °C for 15 s and annealing/extension at 60 °C for 1 min. In the end, the dissociation stage was added to check the specificity of amplicon. The real-time PCR data were analyzed using the 2^−ΔΔ^*^C^*^t^ method.

### 4.8. Transmission Electron Microscopy

After dissection, the eye cups were immediately fixed with pre-chilled 2.5% glutaraldehyde solution (pH 7.4), then treated with 1% osmium tetroxide solution and dehydrated with graded ethanol. The samples were treated with epoxy propane and embedded in 812 resin (Electron Microscopy Sciences, Hatfield, PA, USA). Ultra-thin sections (±50 nm) were prepared by an ultra-microtome (Leica Ultracut-R, Bensheim, Germany). The sections were stained with uranyl acetate and lead citrate and observed under transmission electron microscope (HITACHI-7500, Tokyo, Japan). The pictures were taken by a MegaView digital electron microscopy system (Olympus Soft Imaging Solutions Corp., Lakewood, CO, USA) with appropriate magnifications.

### 4.9. Retinal Cryosections, Immunofluorescence and Fluorescence Microscopy

Five diabetic rats at week 10 after induction were injected in the right eyes as described above with TransIT-TKO^®^ (Mirus Bio LLC, Madison, WI, USA) -treated vector encoding anti-CTGF shRNA and RFP or anti-CTGF shRNA only. One week following injection, the animals were deeply anesthetized with 10% chloral hydrate and thoroughly intracardially perfused with ice-cold PBS, then with 1% PFA (100 mL/kg). Both the injected and un-injected eyes were collected, post-fixed, and dehydrated in graded sucrose solution. The eye balls were embedded in Tissue-Tek^®^ O.C.T. compound (Sakura Finetek, Torrance, CA, USA) and frozen in dry ice, then sagittally sectioned. The sections (5 μm) were either directly observed under fluorescence microscope (BX51, Olympus, Tokyo, Japan) after mounting on the slides with ProLong^®^ Gold Antifade reagent (with DAPI, Life Technologies, Tianjin, China) or subjected to immunofluorescence analysis of RFP as previously described [47,48]. The sections were stained with rabbit anti-RFP antibody (1:3000, Life Technologies, Tianjin, China) at 4 °C overnight, and then detected with Alexa Fluor 596-conjugated secondary antibody (1:400, Abcam, Tianjin, China). All pictures were taken and processed under identical optical parameters by the cellSens Standard electronic system (Olympus, Tokyo, Japan).

### 4.10. Statistics

Data are expressed as the mean ± SE and analyzed by non-paired *t*-test using Statistical Program for Social Sciences 13.0 (SPSS Inc., San Diego, CA, USA). A level of *p* < 0.05 is considered statistically significant.

## 5. Conclusions

In summary, this study for the first time examined the effects of an FDA-approved clinical medication, ranibizumab, and CTGF shRNA in diabetic retinas. The results showed that the combined intervention efficiently normalized the up-regulated expression of both *VEGF* and *CTGF* genes, as well as ameliorated the retinal microvessel ultrastructural changes in STZ-induced diabetic rats. The results of this study suggest the potential of a novel dual-target therapeutic modality to DR.
